# Accurate tree disc volume estimation using TLS: validation and improvement via point cloud repair

**DOI:** 10.3389/fpls.2025.1654386

**Published:** 2025-09-11

**Authors:** Lu Xie, Fangming Wu, Dan Zhao, Liming Du, Jinchen Wu, Cong Xu, Junhua Chen, Xuan Mu, Ping Zhao, Xiaomin Li, Qianhui Zheng, Jinghui Meng, Yuan Zeng, Bingfang Wu

**Affiliations:** ^1^ School of Forestry, Beijing Forestry University, Beijing, China; ^2^ State Key Laboratory of Remote Sensing and Digital Earth, Aerospace Information Research Institute, Chinese Academy of Sciences, Beijing, China; ^3^ University of Chinese Academy of Sciences, Beijing, China; ^4^ Institute of Forest Resources Information Techniques, Chinese Academy of Forestry, Beijing, China; ^5^ Academy of Forestry Inventory and Planning, National Forestry and Grassland Administration, Beijing, China

**Keywords:** terrestrial laser scanning, disc volume estimation, volume accuracy validation, water displacement method, 3D reconstruction

## Abstract

**Introduction:**

Tree trunk volume is a key parameter in forest inventory. Traditional forest surveys typically rely on sample trees and trunk volume equations to estimate tree trunk volume; however, the collection of sample trees is destructive, and trunk volume equations often involve considerable estimation errors. As an emerging technology, terrestrial laser scanning (TLS) has been regarded as an efficient and high-precision alternative for tree trunk volume estimation. Nevertheless, the accuracy of TLS in tree-level trunk volume estimation still lacks systematic evaluation.

**Methods:**

To this end, this study used TLS to scan disc samples cut from standard trees, and evaluated the reliability of TLS-based tree trunk volume estimation by comparing point cloud-derived disc volumes with those obtained using the water displacement method. Utilizing the Leica RTC360 scanner, 123 disc samples from four tree species (*Altingia excelsa, Robinia pseudoacaci, Platycladus orientalis,* and *Quercus suber*) were collected. A novel bottom surface filling algorithm based on point cloud projection was developed to mitigate data loss at disc bases, followed by Poisson surface reconstruction and trunk volume calculation via the Divergence Theorem.

**Results:**

The results demonstrated high accuracy (R² = 0.940, CCC = 0.9745, rRMSE = 14.92%), with a slight underestimation bias (-5.31 cm³). Species-specific analyses indicated significant differences in estimation accuracy (Kruskal-Wallis, H = 21.1606, p = 0.0001), with *Platycladus orientalis* exhibiting the highest accuracy (rRMSE = 4.37%) due to its smooth bark and uniform wood structure, while *Quercus suber* showed the largest errors (rRMSE = 7.10%) attributed to its rough, blocky bark.

**Discussion:**

Bark characteristics and wood structure were identified as key factors influencing TLS accuracy. The analysis revealed that smoother scanned surfaces—comprising both bark surfaces and cross-sections—resulted in higher estimation accuracy. These surface characteristics are closely linked to species-specific external texture and internal wood structure. This study elucidates the influence mechanisms of species-specific physical characteristics on the accuracy of TLS-based trunk volume estimation and proposes targeted strategies for optimizing scanning parameters and point cloud processing. The study provides a robust theoretical and technical foundation for high-precision, non-destructive tree trunk volume measurement in forestry applications.

## 1 Introduction

Forest resource surveys serve as a prerequisite for effective forest management and planning, Sustainable forest resource monitoring and management largely rely on reliable forest inventory data. Tree trunk volume, as one of the key indicators in forest resource inventories, is closely associated with tree biomass, total trunk volume, and numerous ecological traits, enabling a more accurate characterization of tree biomass and various ecological attributes (Zianis et al., 2005). Accurate estimation of trunk volume is crucial for understanding forest resources and carbon storage.

During the process of trunk volume estimation, the complexity of tree trunk morphology makes direct measurement and assessment of trunk volume a challenging task. Typically, trees must be felled, and techniques such as water displacement (i.e., the drainage method) or lignin determination are employed to achieve higher precision in determining individual tree trunk volume (Filho and Schaaf, 1999). Compared to felling trees to obtain trunk volume, empirical models based on measurable parameters, such as diameter at breast height (DBH) and tree height, offer greater flexibility in trunk volume estimation. Among traditional methods,taper Equation due to their accuracy and high adaptability, have become the standard method for estimating tree trunk volume. Modern taper equations can be classified into four main types: variable-form taper equations, compatible taper equations, polynomial, segmented, and spline taper equations, as well as trigonometric and matrix taper equations (McTague and Weiskittel, 2021).

Although taper equations are widely applied in forest mensuration, they are subject to several limitations. Most highly flexible variable-form equations entail significant computational demands, particularly in the context of large-scale forest resource inventories (Kozak, 2004; McTague and Weiskittel, 2021). Most taper equations rely on measured diameter at breast height (DBH) and tree height as primary covariates, while additional variables such as crown ratio or upper stem diameter may also be required. Measurement errors in these variables can lead to significant biases in calculations, particularly for tree height, as accurate height estimation remains a persistent challenge in forest resource inventories. Typically, forest inventories measure tree height for only a subsample (5%–30%), with the remaining values estimated using regionally or locally calibrated equations. The inaccuracies in individual tree trunk volume estimates can substantially amplify when aggregated to the stand or larger scales (McTague and Weiskittel, 2021).

Over the past two decades, LiDAR technology has introduced significant breakthroughs in forest resource inventories by providing precise three-dimensional information (Fan et al., 2023; He et al., 2025; Lovell et al., 2003; McTague and Weiskittel, 2021; Wang et al., 2021). Understory LiDAR technology, capable of capturing detailed structural information of forests from beneath the canopy, provides a high-precision method for tree trunk volume measurement. Currently, the most widely applied types of understory scanning LiDAR in forest resource monitoring include stationary Terrestrial Laser Scanners (TLS) and Mobile Laser Scanners (MLS) (Zhang et al., 2024).

TLS are mounted on fixed ground platforms, such as tripods, to conduct multi-station scanning (Bauwens et al., 2016), Due to its stable platform positioning, the terrestrial laser scanner achieves the highest point cloud density and scanning accuracy (Liang et al., 2015). TLS has been widely applied in forest resource inventories, particularly for assessing forest tree attributes such as diameter at breast height (DBH), tree height, and tree location (Bienert et al., 2006; Maas et al., 2008). TLS has also been extensively utilized for the estimation of trunk volume and biomass. Liu et al. (2018) (Liu et al., 2018) estimated DBH (average Root Mean Square Error (RMSE) = 1.52 cm) and tree height (average RMSE = 0.95 m) for four tree species using TLS point cloud data obtained in Diqing, Yunnan.

Owing to the flexibility of MLS, numerous studies have explored its applications in forestry. D’hont et al. (2024) (D’hont et al., 2024) evaluated the performance of TLS and MLS in estimating DBH and tree height under both leaf-on and leaf-off conditions. Their study indicated that TLS provides the most accurate DBH measurements, with the lowest RMSE (RMSE = 0.019 m), establishing it as the benchmark method for DBH estimation. MLS performed better under leaf-off conditions but was slightly less accurate during leaf-on conditions. For tree height estimation, TLS was also the optimal choice (RMSE = 0.357 m), while MLS achieved comparable accuracy to TLS only under leaf-off conditions (RMSE = 0.37 m). In terms of efficiency, MLS does not require multi-station scanning, enabling surveyors to achieve an average coverage rate of 50 m²/min. This is significantly higher than the 0.85 m²/min of TLS instruments and the 0.43 m²/min of field surveys, markedly improving the efficiency of forest resource monitoring (Ryding et al., 2015). These studies collectively demonstrate that, compared to field measurements for obtaining DBH and tree height, MLS-based estimations can achieve high accuracy while substantially enhancing field survey efficiency due to its flexibility.

Although MLS offers high efficiency, high-precision multi-station TLS scanning remains the optimal choice when pursuing maximum accuracy and conducting method validation (Duncanson et al., 2019). While numerous studies have validated the accuracy of trunk volume estimation based on TLS data, these studies typically use measured DBH and tree height as parameters, calculating trunk volume through empirical models such as taper equations to serve as reference values (Moskal and Zheng, 2011; Pueschel et al., 2013; Thies et al., 2004). However, the accuracy of field-measured tree heights often exhibits significant errors, which directly impacts the reliability of trunk volumes derived from these measurements (Wang et al., 2018). Consequently, current research lacks validation and evaluation of TLS trunk volume estimation accuracy using directly measured trunk volumes as true reference values.

Therefore, this study utilizes TLS scanning to measure discs cut from standard trees, comparing the disc trunk volumes calculated from point cloud data with those obtained via the water displacement method. It specifically investigates and addresses the following questions: (1) How to mitigate estimation errors caused by missing point cloud data at the bottom of disc scans; (2) How precise is trunk volume estimation based on terrestrial LiDAR; (3) Whether tree species characteristics affect the accuracy of LiDAR scanning, and which specific factors influence this accuracy.

## 2 Materials and methods

### 2.1 Study area

**Figure 1:**
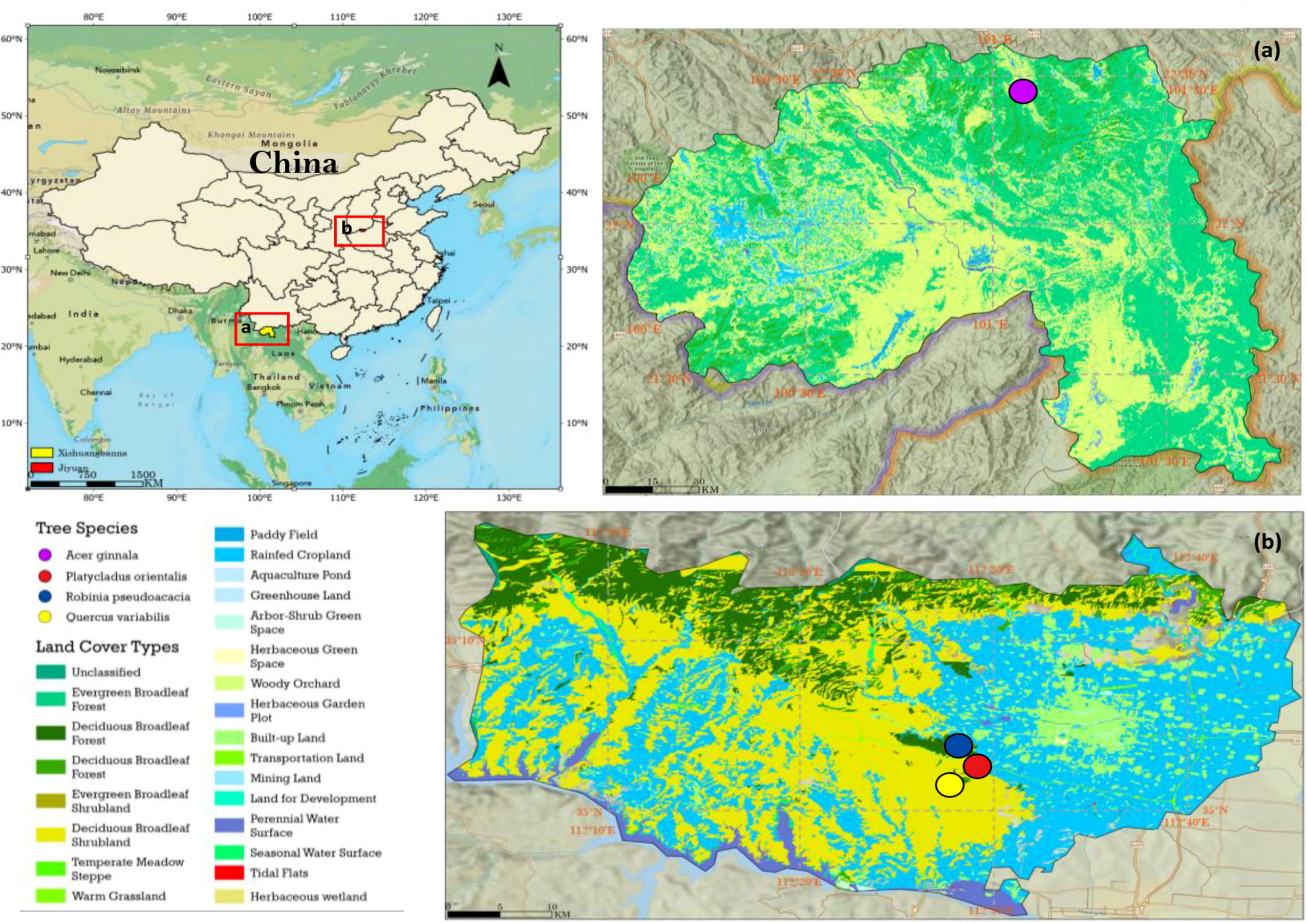
Location maps and land cover classification of the study areas. (Left) Overview map of China showing the locations of two study sites: **(a)** Jinghong City, Xishuangbanna Dai Autonomous Prefecture, Yunnan Province; and **(b)** Xiaolangdi Town, Mengjin District, Luoyang City, Henan Province. (Right-top, a) Land cover classification map of the Xishuangbanna site; (Right-bottom, b) Land cover classification map of the Xiaolangdi site. Colored dots represent the locations of sample plots with different tree species. Land cover data were derived from the ChinaCover 16 m land cover dataset.

The study area is located in Puwen, Yunnan, and Xiaolangdi, Henan, China (**Figure 1**), with the primary forest types being subtropical evergreen broad-leaved forest and warm-temperate deciduous broad-leaved forest, respectively, forming a comparative research framework across climatic zones. The Puwen study area in Yunnan is situated in Jinghong City, Xishuangbanna Dai Autonomous Prefecture, Yunnan Province ( 101°01'13″−101°06'02″E, 22°28'20″−22°33'08″N ) (**Figure 1a**). The terrain is undulating, with elevations ranging from 847 m to 1,285 m, and an average elevation of approximately 972 m. The region exhibits a typical tropical monsoon climate with distinct dry and wet seasons, an annual average precipitation of 1,347.4 mm, primarily concentrated in the rainy season, and an annual average temperature of 22.9°C. The area has a high forest coverage rate, with vegetation dominated by tropical and subtropical evergreen broad-leaved forests. The region is characterized by rich biodiversity, with *Altingia excelsa* being a dominant tree species of significant ecological and economic value. The Xiaolangdi study area in Henan is located in Xiaolangdi Town, Mengjin District, Luoyang City, Henan Province ( 112°14"−112°30"E, 34°46"−35°05"N ) (**Figure 1b**). The terrain is markedly undulating, with elevations ranging from 500 m to 1,300 m. The region features a warm-temperate contin ental monsoon climate with distinct seasons, an annual average precipitation of approximately 600 mm, and an annual average temperature of about 14 °C. The vegetation is predominantly warm-temperate deciduous broad-leaved forest, forming a multi-layered forest ecosystem structure. The main tree species include *Robinia pseudoacacia, Paulownia* spp.*, Platycladus orientalis, Sophora japonica, and Salix matsudana*, while the shrub layer primarily consists of *Amorpha fruticosa* and *Vitex negundo.*

### 2.2 Data acquisition

#### 2.2.1 Sample collection

This study first required the selection and felling of sample trees to produce standard logs. During the sample tree selection process, priority was given to individuals with high stem straightness, healthy growth conditions, and typical morphological characteristics, while trees with evident pest, disease, or mechanical damage were excluded. To ensure data representativeness and comparability, the diameter at breast height (DBH) of sample trees was controlled within a range of 15–45 cm, with an emphasis on covering the main tree species types and diverse diameter classes in the study areas to comprehensively reflect regional characteristics. Based on these criteria, 16 valid sample trees were ultimately selected for subsequent analysis. These samples included 10 *Altingia excelsa trees* from the Puwen area and 3 *Robinia pseudoacacia*, 1 *Platycladus orientalis*, and 3 *Quercus suber trees* from the Xiaolangdi area.

**Figure 2:**
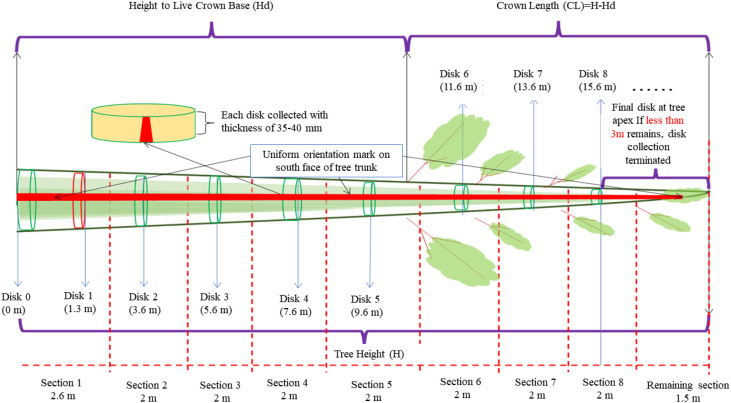
Schematic diagram of discs extraction from the tree stem. Discs of 35–40 mm thickness were sampled at specified heights from the stem base to the apex. A reference line was inscribed on the southern face of the trunk to maintain consistent orientation. Sampling was concluded below the tree apex, with collection terminated if the final apical section was less than 3 m in length. (H, tree height; Hd, height to live crown base; CL, crown length).

Field data were collected in the spring and autumn of 2023, with sampling for each tree species conducted within the same time period to minimize potential impacts of seasonal variations on sample characteristics, ensuring temporal consistency and scientific comparability of the data. The sampling process adhered to standardized operational procedures. Initially, selected sample trees were felled at ground level to ensure sample integrity. Subsequently, systematic trunk sampling was performed, with initial cross-sectional cuts made at 0.3 m (base), 1.3 m (diameter at breast height), and 2.6 m above the ground, followed by longitudinal segmentation at 1-meter intervals along the trunk to construct a comprehensive trunk profile sampling system (**Figure 2**). During sampling, disc thickness was strictly controlled within a range of 5 ± 0.5 cm to ensure consistency in physical characteristics across samples. All disc samples were labeled on non-working surfaces with complete identification information, including plot number, tree number, and sampling height, establishing a rigorous sample traceability system.

#### 2.2.2 TLS LiDAR data acquisition and preprocessing

**Figure 3:**
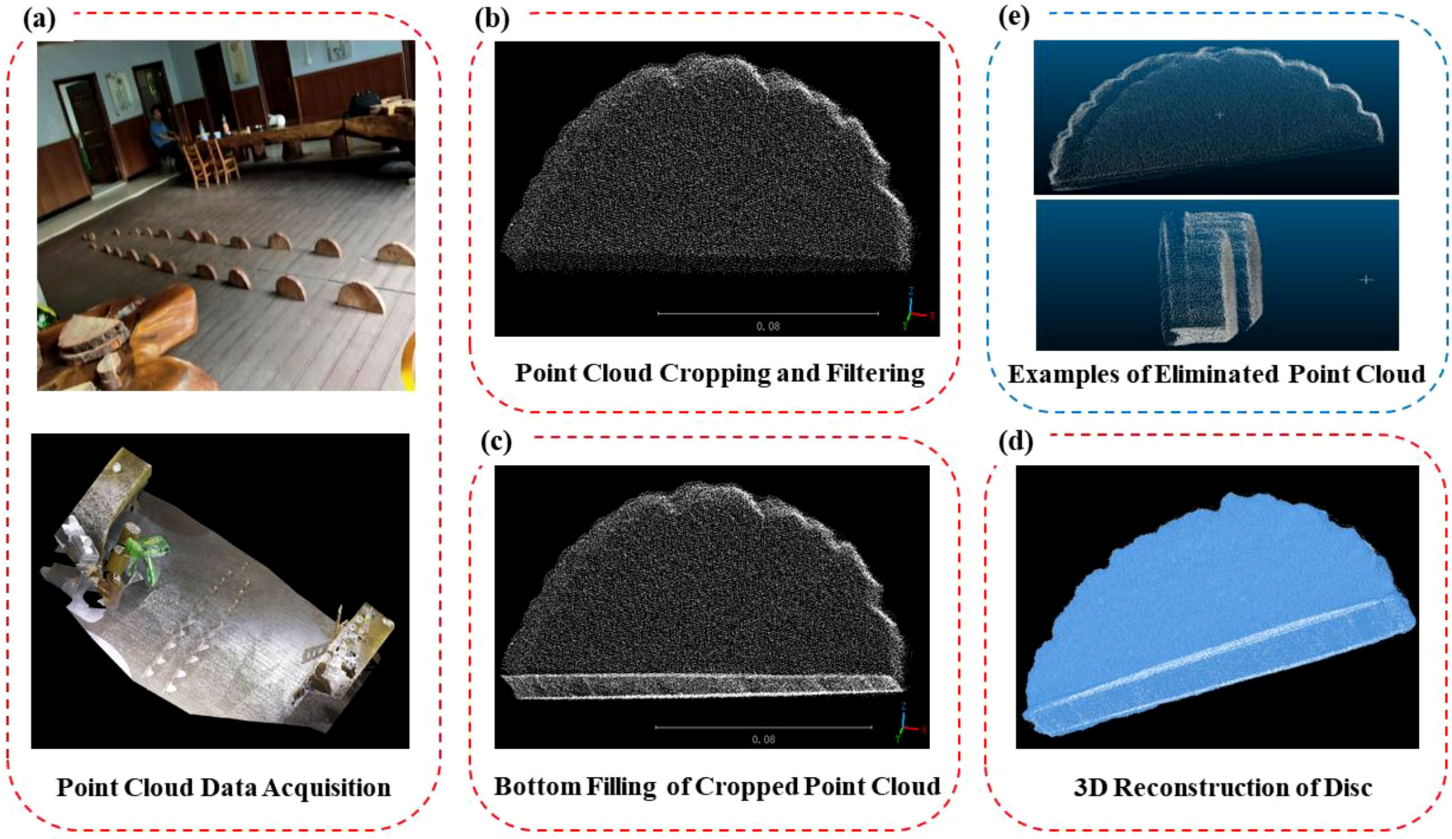
Schematic diagram of point cloud data processing and filtering. **(a)** Point Cloud Acquisition **(b)** Point Cloud Cropping and Filtering **(c)** Bottom Filling of Cropped Point Cloud **(d)** 3D Reconstruction of Disc **(e)** Examples of Eliminated Point Cloud.

This study utilized the Leica RTC360, a high-speed terrestrial laser scanner with a maximum scanning range of 130 m, a ranging error of ±1.0 mm, and an angular accuracy of 18″. It offers advantages such as ultra-high-speed scanning, high-precision ranging, compatibility with multiple point cloud formats, and integration with mainstream modeling software. All disc samples were subjected to three-dimensional scanning, with the scanning process strictly controlled to maintain an angular resolution of 0.02°, a point cloud density of at least 1000 points/dm², and a standard scanning distance of 3 m. The disc samples from each tree were arranged in the order of cutting as shown in **Figure 2**, and placed sequentially as illustrated in **Figure 3a**), and scanning was conducted using a three-station layout positioned in the northeast, southeast, and west directions to ensure comprehensive coverage and minimize shadowing effects. Marker-assisted registration was employed during scanning to align data from different scan positions. The acquired point cloud data underwent multiple preprocessing steps (**Figure 3**), primarily including automatic point cloud registration and manual noise removal using CloudCompare’s rectangular selection and cropping tools. Following preprocessing, the point cloud was further refined through detailed segmentation to effectively extract the disc point cloud while eliminating interference from background elements and supporting structures.

#### 2.2.3 Water displacement method data acquisition

**Figure 4:**
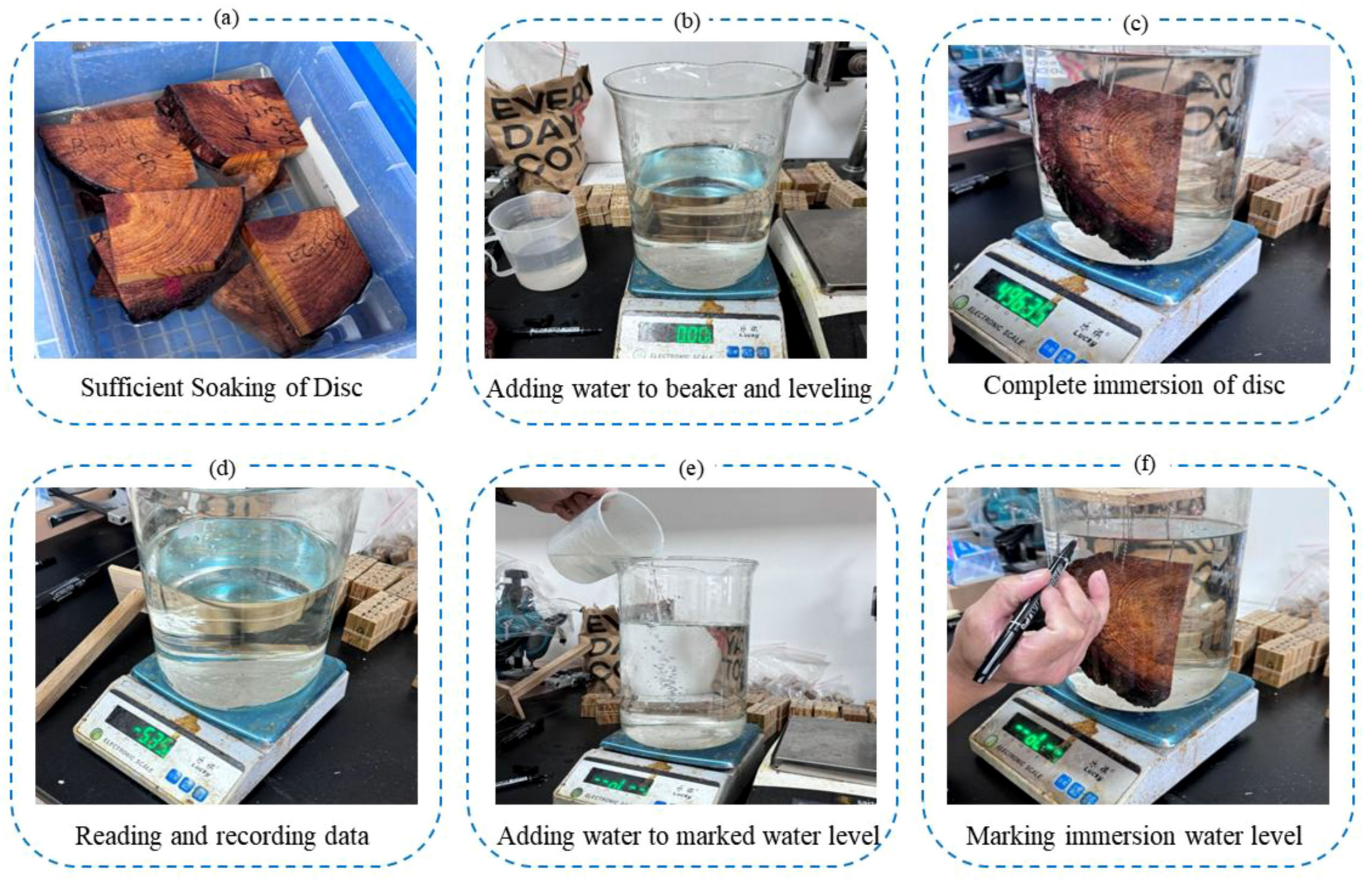
Workflow of disc volume measurement using the water displacement method. **(a)** Sufficient Soaking of Disc **(b)** Adding water to beaker and leveling **(c)** Complete immersion of disc **(d)** Reading and recording data **(e)** Adding water to marked water level **(f)** Marking immersion water level.

This study adopts the disc volume measured by the water displacement method, as specified in the national standard GB/T 1927.5-2021, as the true disc volume of the discs (Martin, 1984). To ensure measurement precision, a standard graduated cylinder with an accuracy of 0.1 ml was used. The measurement process followed standardized operational procedures (**Figure 4**): First, an appropriate amount of distilled water was poured into the cylinder, and the initial water level was precisely recorded. Subsequently, the sealed or thoroughly soaked disc sample was fully submerged in the water, ensuring no air bubbles adhered to the sample surface. After the water level stabilized, the final water level was accurately recorded. The actual disc volume was calculated based on the displaced water volume (the difference between the final and initial water levels). To reduce random errors and enhance data reliability, three independent measurements were conducted for each disc sample, and the arithmetic mean of the three measurements was used as the final reference trunk volume value for the sample.

#### 2.2.4 Disc data filtering

All collected data underwent a rigorous quality control filtering process. The filtering criteria primarily included: (1) Ensuring the integrity of point cloud data by excluding samples with incomplete scans or significant occlusion. For instance, some point clouds exhibited ghosting due to improper operation during scanning, and some discs contained voids, which could lead to substantial errors in subsequent three-dimensional trunk volume reconstruction. (2) Ensuring the reliability of validation data by eliminating samples with anomalies during the water displacement measurement process. For example, some discs with excessively large diameters were cut into smaller pieces for trunk volume measurement during the water displacement process, resulting in unreliable validation data. Following this series of stringent screening steps, 123 samples meeting the quality requirements were ultimately selected from the initial sample pool for subsequent analysis (**Table 1**).

**Table 1:** Summary of sample sizes after filtering.

Species	Sample size	Average disc volume (cm³)
*Altingia excelsa*	69	146.78
*Robinia pseudoacacia*	25	205.99
*Platycladus orientalis*	12	148.62
*Quercus suber*	17	236.68
total	123	171.42

### 2.3 Point cloud processing and 3D reconstruction

Existing TLS-based tree volume reconstruction methods, such as TreeQSM, AdQSM (Ali et al., 2025; Fan et al., 2020; Hackenberg et al., 2015), and SimpleForest, generally approximate the stem as a sequence of cylindrical components. However, due to the complexity and variability of actual stem morphology during growth, such simplifications may introduce estimation errors. To address this, the present study focuses on a finer-scale validation at the cross-sectional level to assess the accuracy of TLS-derived stem volume estimates.Due to the placement of discs on the ground during scanning, data from the bottom surface of the disc samples were missing.

To address this, this study developed a bottom surface filling algorithm based on the point cloud projection principle. The point cloud of each disk was projected from the Z-axis onto the XY-plane to compensate for the missing bottom surface caused by ground contact during scanning. This projection enabled the reconstruction of a complete disk point cloud, facilitating subsequent 3D modeling. The process begins with lowest point detection, where the three-dimensional point cloud data structure is analyzed through precise scanning to identify the minimum value along the *Z*-axis (Equation 1)a)i)i), denoted as *Z_min_*, which represents the global minimum *Z*-coordinate (height) of all points. Here, *Z_i_* denotes the *Z*-coordinate of the *i* ^th^ point in the original point cloud P, and this minimum Z-value plane serves as the reference baseline.


(1) $$\begin{array}{c} z_{min}={\text{min}}_{i=1}^{n}z_{i} \end{array}$$

Thereafter, vertical projection is performed on the reference plane. All original point clouds are duplicated, retaining their positions on the *XY* axes unchanged, while their *Z*-coordinates are set to the minimum *Z*-value obtained during lowest point detection (Equation 2). Here, *P_i_* represents the three-dimensional coordinates of the *i* ^th^ point in the original point cloud, denoted as $\left(x_{i},y_{i},z_{i}\right)$ , where $x_{i},y_{i},$ are the horizontal coordinates, and $z_{i}$ is the height. The transformed coordinates of the *i* ^th^ point after applying the above method are denoted as $p_{i}^{'}$ .


(2) $$\begin{array}{c} p_{i}^{'}=T(p_{i})=(x_{i},y_{i},z_{min}) \end{array}$$

To complete the process, point cloud merging is conducted, where the ground point cloud generated through projection (Equation 3) is precisely integrated with the originally collected point cloud data (Equation 4)to construct a complete three-dimensional surface model of the sample. Here, $P_{\text{projected}}$ denotes the set of projected points, and $P_{\text{complete}}$ represents the complete point cloud data set.


(3) $$\begin{array}{c} P_{\text{projected}}=\{(x_{i},y_{i},z_{\text{min}})|(x_{i},y_{i},z_{i})\in P_{\text{original}}\} \end{array}$$

(4) $$\begin{array}{c} P_{\text{complete}}=P_{\text{original}}\cup P_{\text{projected}} \end{array}$$

Once the complete point cloud was generated, normal vector calculations were performed on the complete point cloud. This study utilized CloudCompare software for normal vector computation, selecting a quadratic surface fitting model suitable for surfaces with high curvature. Initially, a local neighborhood search was conducted for each point to construct a local point cloud subset comprising the target point and its six nearest neighbors. A quadratic surface equation was then fitted to this subset using the least squares method, where.

(x,y,z)  represents the local coordinates of the point cloud, and a,b,c,d,e,f are the fitted quadratic surface parameters (Equation 5).


(5) $$\begin{array}{c} z=ax^{2}+by^{2}+cxy+dx+ey+f \end{array}$$

Normal vectors were calculated based on the surface gradient principle. The partial derivatives of the quadratic surface with respect to the *X* and *Y* directions were computed to obtain the gradient direction, which was then normalized to yield the normal vector (Equation 6). To ensure directional consistency across topologically adjacent regions, the Minimum Spanning Tree (MST) algorithm was applied to globally optimize the normal vector directions, ensuring their consistency across topologically adjacent regions.


(6) $$\begin{array}{c} N=(-\frac{\partial z}{\partial x},-\frac{\partial z}{\partial y},1) \end{array}$$

For trunk volume estimation, a method combining Poisson reconstruction and the divergence theorem was employed for trunk volume estimation. Building on the preceding research, the point cloud $P_{\text{complete}}$ was processed in CloudCompare software using the Poisson surface reconstruction method to generate a closed triangular mesh model. Initially, normal vectors $n_{i}$ were estimated for each point based on the local neighborhood geometry of the point cloud, using covariance analysis with *k*=8 nearest neighbors to compute the normal vectors. Subsequently, the points and their normal vectors were integrated into a vector field (Equation 7), where w(p) represents a weight function based on point density.


(7) $$\begin{array}{c} V(i)=w(i)n_{i} \end{array}$$

Next, the Poisson equation (Equation 8) was solved using Fast Fourier Transform to generate an indicator function, which defines the internal and external boundaries of the point cloud.


(8) $$\begin{array}{c} \Delta \chi =\nabla \cdot V \end{array}$$

To extract the surface, the Marching Cubes algorithm was employed to extract the zero isosurface at χ=0 generating a continuous and closed triangular mesh model, ensuring complete surface coverage of the disc.

Based on the closed mesh, the trunk volume of the tree disc was calculated using the Divergence Theorem, which transforms a trunk volume integral into a surface integral. By summing the trunk disk sample contributions of each triangular face on the closed surface and multiplying by a coefficient of 1/3, the precise trunk volume of the disc sample was obtained (Equation 9). where $A_{i}\;$ represents the area of the *i* ^th^ triangle, $p_{i}\;$ denotes the centroid of the *i* ^th^ triangle, and $n_{i}$ is the normal vector of the *i* ^th^ triangle. The term $p_{i}\cdot n_{i}$ indicates the projection length of the centroid along the normal vector, with $A_{i}$ providing the area weighting.


(9) $$\begin{array}{c} V=\frac{1}{3}\sum_{i}A_{i}(p_{i}\cdot n_{i}) \end{array}$$

### 2.4 Statistical analysis

To evaluate the correlation between tree trunk volume estimates derived from terrestrial laser scanning and actual trunk volumes measured using the water displacement method, this study employed multivariate statistical analysis to establish a linear model between the two methods. To evaluate the accuracy of trunk volume estimation using terrestrial laser scanning (TLS) compared to the water displacement method, regression equations and the coefficient of determination (R²) were calculated. Scatter plots, overlaid with an identity line and a regression line, were used to visually illustrate the correlation and degree of deviation between the two methods. For accuracy evaluation, four metrics were comprehensively calculated: Root Mean Square Error (RMSE), Relative Error (RE), Bias, and Relative Root Mean Square Error (rRMSE), to thoroughly analyze both absolute and relative differences in measurement accuracy.

However, linear correlation metrics (such as the coefficient of determination) only reflect the strength of the linear relationship between variables and are not suitable for assessing the consistency of measurement methods (Giavarina, 2015). Thus, this study employed the Bland-Altman method (Mansournia et al., 2020). The core of the Bland-Altman method lies in quantifying the agreement between two methods by analyzing the differences between their measurements, rather than relying on their correlation. Specifically, it calculates the difference for each pair of measurements and plots these differences against the mean of the two measurements in a scatter plot (known as a Bland-Altman plot). The mean difference, $\bar{d}$ , indicates whether one method systematically overestimates or underestimates the other; for instance, a $\bar{d}$ close to zero suggests minimal bias. The limits of agreement, defined as $\bar{d}\pm 1.96\times s$ , where *s* is the standard deviation of the differences, encompass 95% of the differences. A narrower range indicates closer agreement between the two methods, reflecting higher consistency. Additionally, the plot visually reveals whether differences vary with the magnitude of the measurements, aiding in evaluating the performance of the two methods across different measurement ranges (Giavarina, 2015). Meanwhile, the Concordance Correlation Coefficient (CCC) was calculated to assess the strength of agreement between the measurement results. This method has been widely applied in dual-measurement comparison studies, such as those in forest stand trunk volume estimation. The interpretations of the aforementioned statistical metrics are presented in**Table 2**.

**Table 2:** Description of statistical metrics.

Indicator name	Calculation formula	Description
Coefficient of Determination	$\begin{array}{c} R^{2}=1-\frac{\sum {\left(y_{i}-x_{i}\right)}^{2}}{\sum {\left(y_{i}-\bar{y}\right)}^{2}} \end{array}$	The ratio of explained variance to total variance, measuring the proportion of variation explained by the model.
Root Mean Square Error	$\begin{array}{c} \text{RMSE}=\sqrt{\frac{1}{n}\sum_{i=1}^{n}{\left(y_{i}-x_{i}\right)}^{2}} \end{array}$	the square root of the average squared differences between predicted and observed values.
Relative Error	$\begin{array}{c} \text{RE}=\frac{1}{n}\sum_{i=1}^{n}\frac{x_{i}-y_{i}}{y_{i}} \end{array}$	the average of relative differences between predicted and observed values, expressed as a proportion.
Bias	$\begin{array}{c} \text{Bias}=\frac{1}{n}\sum_{i=1}^{n}(y_{i}-x_{i}) \end{array}$	the average difference between observed and predicted values indicating systematic over- or under-prediction.
Relative Root Mean Square Error	$rRMSE=\frac{RMSE}{\bar{y}}\times 100\%$	the ratio of RMSE to the mean of observed values, providing a normalized measure of error.
Upper Limit of Agreement (ULOA)	$\begin{array}{c} \text{ULOA}=\bar{d}+1.96\cdot SD_{d} \end{array}$	the upper boundary of the 95% confidence interval for the difference between measurement methods.
Lower Limit of Agreement (LLOA)	$\begin{array}{c} \text{LLOA}=\bar{d}-1.96\cdot SD_{d} \end{array}$	the lower boundary of the 95% confidence interval for the difference between measurement methods.
Concordance Correlation Coefficient (ρc)	$\begin{array}{c} {\text{ρ}}_{c}=\frac{2{\text{ρσ}}_{x}{\text{σ}}_{y}}{{\text{σ}}_{x}^{2}+{\text{σ}}_{y}^{2}+{\left({\text{μ}}_{x}-{\text{μ}}_{y}\right)}^{2}} \end{array}$	a measure combining precision and accuracy to assess agreement between two variables.
Gauge Repeatability and Reproducibility (GRR)	$\begin{array}{c} \text{GRR}=\frac{{\text{σ}}_{\text{GRR}}^{2}}{{\text{σ}}_{\text{total}}^{2}}\times 100\% \end{array}$	the percentage of total variation attributable to the measurement system.
Kruskal-Wallis Test Statistic (H)	$\begin{array}{c} H=\frac{12}{N(N+1)}\sum_{i=1}^{k}\frac{R_{i}^{2}}{n_{i}}-3(N+1) \end{array}$	a non-parametric statistical test used to determine if there are statistically significant differences between three or more groups of samples.
Mann-Whitney U Test Statistic	$\begin{array}{c} U=n_{1}n_{2}+\frac{n_{1}(n_{1}+1)}{2}-R_{1} \end{array}$	a non-parametric test for assessing whether two independent samples come from the same distribution.

To analyze the impact of tree species on measurement accuracy, a difference analysis was conducted using tree species as the grouping variable. Since the sample data did not follow a normal distribution, the Kruskal-Wallis test was chosen to compare the overall distribution of measurement differences among different tree species. Based on significant results, pairwise comparisons were further conducted using the Mann-Whitney U test to identify specific species combinations exhibiting differences. All statistical tests were performed with a significance level of α = 0.05. All analyses were implemented in a Python environment.

## 3 Results

### 3.1 Statistical analysis of overall estimation accuracy

**Figure 5:**
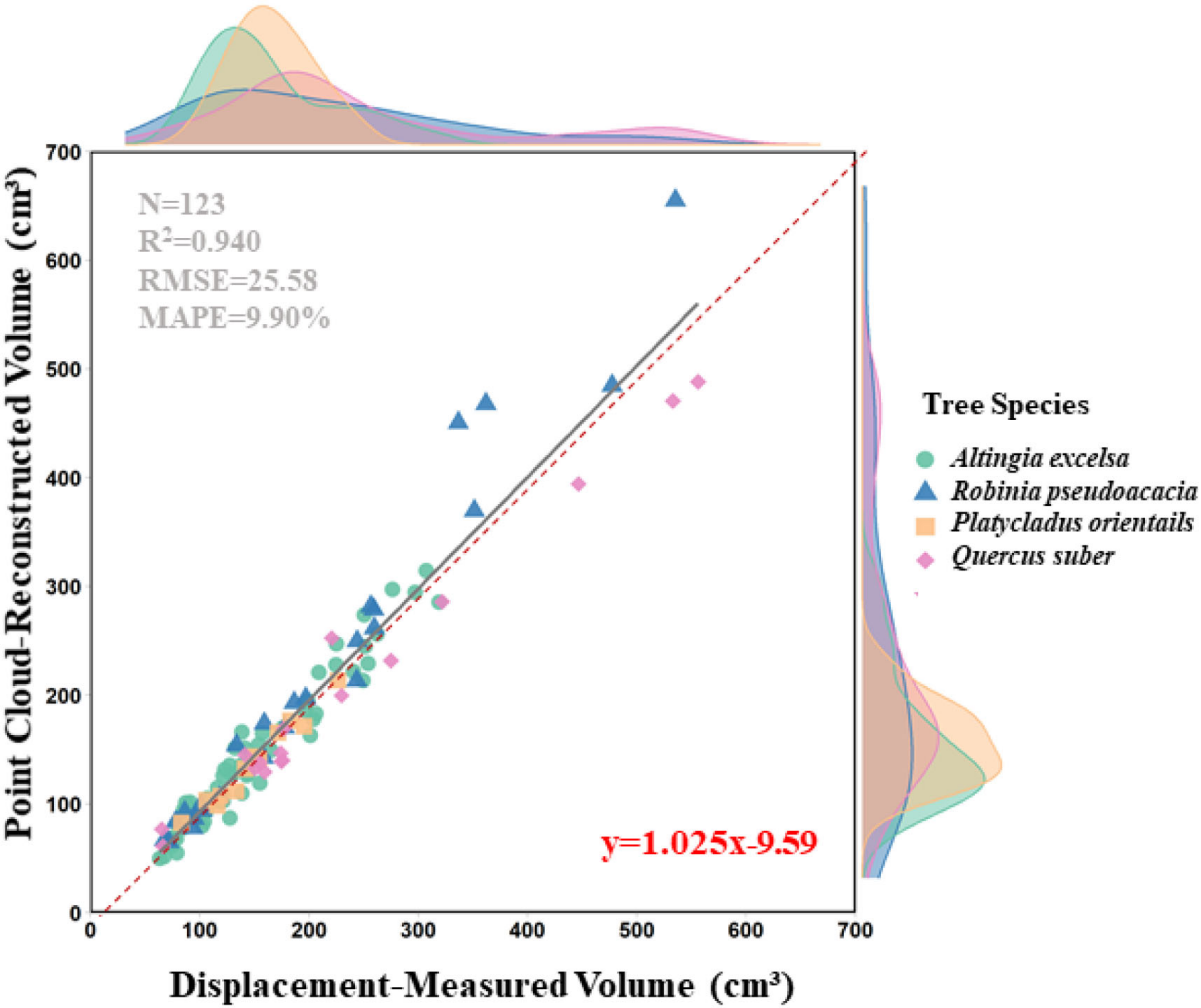
Relationship between point cloud-reconstructed disc volume and displacement-measured disc volume for all disc samples. The solid black line represents the fitted regression line, while the red dashed line indicates the 1:1 line. Different colors and shapes denote different tree species, N: sample size.

Based on 123 measured samples, regression analysis and error evaluation results indicate high accuracy in disc volume estimates derived from terrestrial laser scanning (R² = 0.940,CCC = 0.9745,rRMSE = 14.92%) (**Figure 5**). These metrics provide complementary insights by evaluating the results from multiple dimensions—including goodness of fit, error magnitude, consistency, and measurement system stability—thus enabling a comprehensive assessment of the accuracy and reliability of the trunk volume estimation. The regression analysis reveals that the linear relationship between laser scanning measurements (*y*) and water displacement measurements (*x*) closely approximates a 1:1 relationship, indicating a high degree of consistency between the two methods. Two key insights were derived. First, a slope less than 1 suggests a slight systematic underestimation by the laser scanning method, particularly for larger trunk volume samples. Second, a positive intercept indicates a slight overestimation when measuring smaller trunk volume samples. The Gauge Repeatability and Reproducibility (GRR) value from the Measurement System Analysis (MSA) was 26.07%, which evaluates the repeatability and reproducibility of the measurement system, reflecting the variability in repeated measurements under identical conditions.

**Figure 6:**
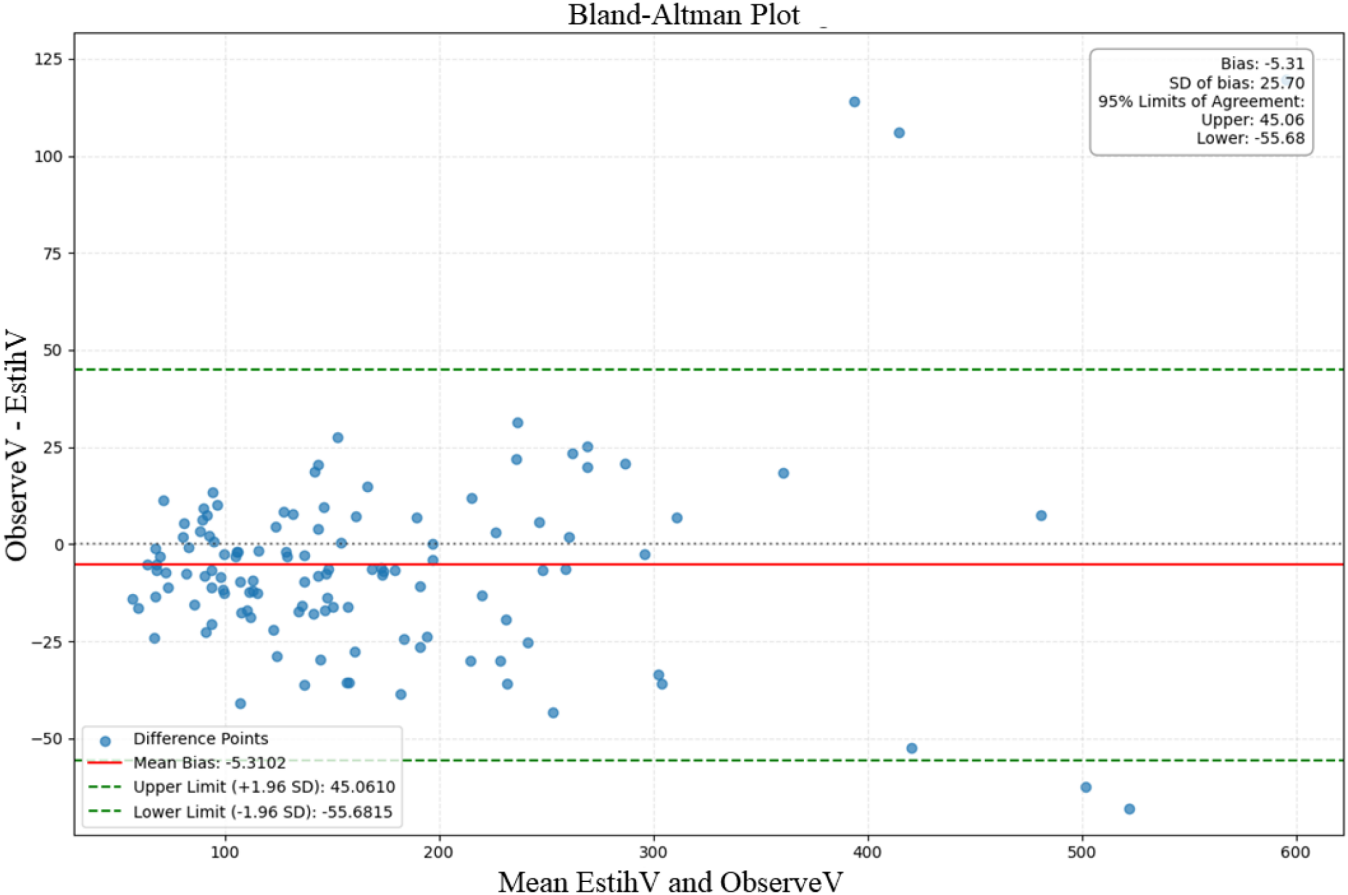
Bland–Altman plot for all samples, showing the agreement between the point cloud-estimated disc volume (EstiV) and the displacement-measured disc (ObserveV). The vertical axis represents the difference between ObserveV and EstiV, while the horizontal axis represents their mean. The red solid line indicates the mean bias, and the green dashed lines represent the 95% limits of agreement (LoA). Blue dots denote individual sample differences.

The disc volume estimates obtained from terrestrial laser scanning were, on average, 5.31 cm³ lower than the reference values, indicating a slight negative bias, with 95% limits of agreement ranging from -55.68 to 45.06 cm³ and a standard deviation of 25.70 cm³ (**Figure 6**). The data points exhibited a funnel-shaped distribution, suggesting that as the measured trunk volume increased (particularly beyond 400 cm³), the consistency between the two methods decreased, with larger differences observed. Overall, the two measurement methods showed good agreement for smaller trunk volume ranges (<300 cm³), but several notable outliers were observed for larger trunk volumes.

### 3.2 Analysis of trunk volume estimation accuracy for different tree

Based on the linear relationships between the laser-scanned trunk volume estimates and the water displacement method for the four tree species (**错误!未找到引用源。**)), a significant linear correlation was observed across all species, with slopes close to 1 and relatively small intercepts, indicating a high degree of consistency between predicted trends and actual observed values. *Altingia excelsa* exhibited a good fit (R² = 0.949), with a slope very close to 1. *Robinia pseudoacacia* showed a higher coefficient of determination (R² = 0.965), but its slope (1.206) was notably greater than 1, with a larger intercept (-27.862), suggesting a potential systematic overestimation in the high-trunk volume range. *Platycladus orientalis* had a coefficient of determination of 0.969, with a slope slightly below the ideal value. Although *Quercus sube*r achieved the highest coefficient of determination (R² = 0.949), its slope was significantly lower, indicating a clear underestimation trend in the high-trunk volume range.

From the perspective of prediction error, *Platycladus orientalis* exhibited the smallest root mean square error (RMSE) of 6.49, indicating overall minimal absolute deviations in predictions. This was followed by *Altingia excelsa* (RMSE = 15.01) and *Quercus suber* (RMSE = 16.81), while *Robinia pseudoacacia* had the largest RMSE (28.80).

In terms of trunk volume estimation accuracy as measured by rRMSE, *Platycladus orientalis* performed best, with an rRMSE of only 4.37%. This outstanding performance is likely attributable to its straight and regular trunk shape, which provides ideal conditions for laser scanning trunk volume estimation. *Quercus suber* followed closely, also exhibiting excellent performance (rRMSE = 7.10%), further demonstrating the effectiveness of this method for tree species with regular trunk structures in temperate forests. *Altingia excelsa* from the subtropical Puwen region in Yunnan showed moderate accuracy (rRMSE = 10.23%), while *Robinia pseudoacacia* had the highest error rate among the studied species (rRMSE = 13.98%).

**Figure 7:**
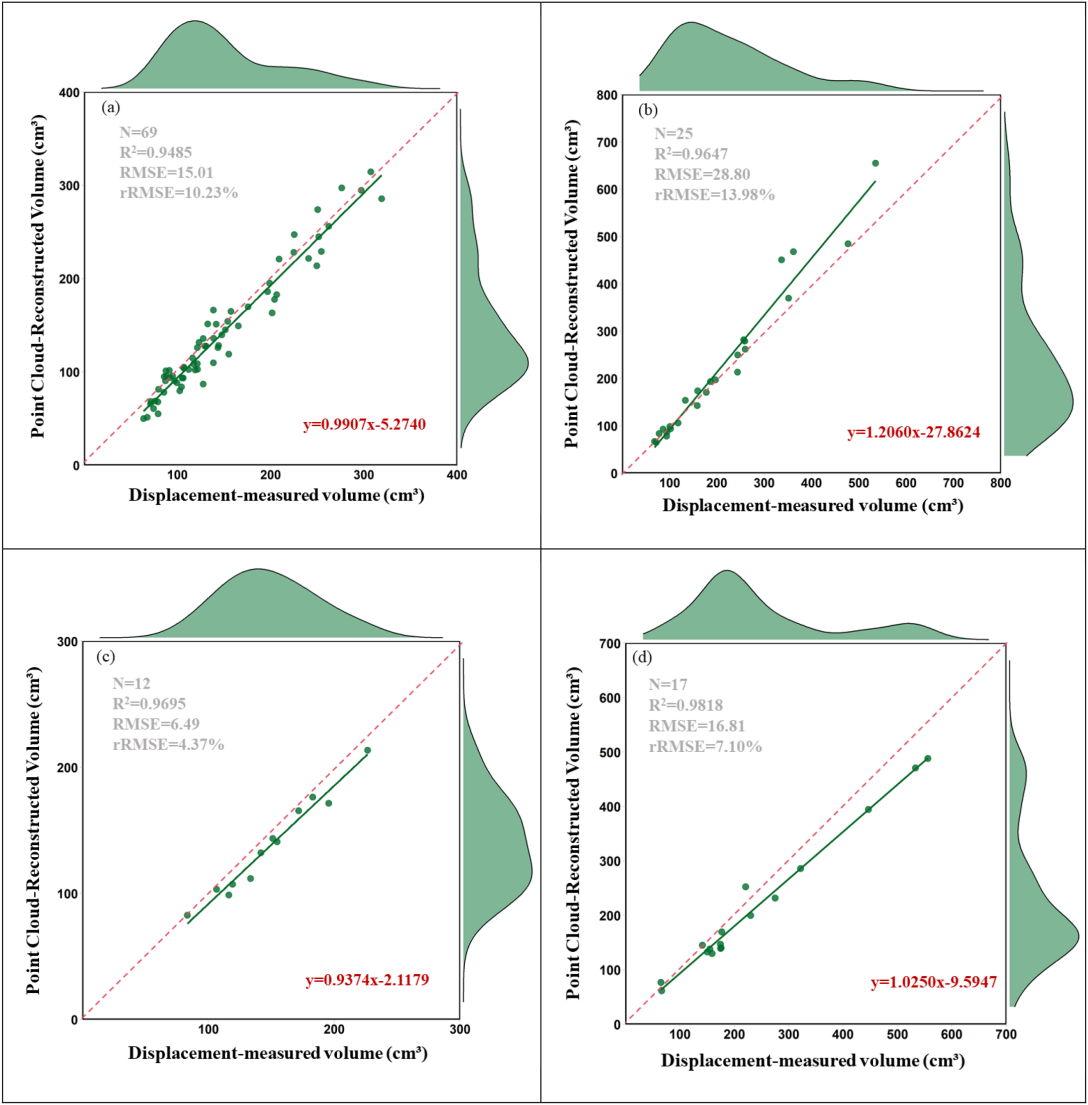
Comparison of point cloud-reconstructed trunk volume and water displacement-measured volume for four tree species: **(a)** *Altingia excelsa*, **(b)** *Robinia pseudoacacia*, **(c)** *Platycladus orientalis*, **(d)** *Quercus suber*. Each panel shows a linear regression line (red), 1:1 reference line (dashed), and kernel density distributions. N: sample size.

Synthesizing all metrics, despite a slight but consistent underestimation trend, *Platycladus orientalis* exhibited the highest overall accuracy in trunk volume estimation. *Altingia excelsa* followed, with an accurate estimation model but a minor underestimation bias. The trunk volume estimates for *Robinia pseudoacacia* showed no statistically significant difference from actual values but displayed greater variability, necessitating caution regarding its overestimation tendency in practical applications. Although *Quercus suber* demonstrated strong statistical fit, it exhibited a pronounced underestimation issue, particularly for larger-trunk volume trees. These findings provide valuable reference for the application of terrestrial laser scanning technology in trunk volume estimation across different tree species (**Figure 7**).

**Figure 8:**
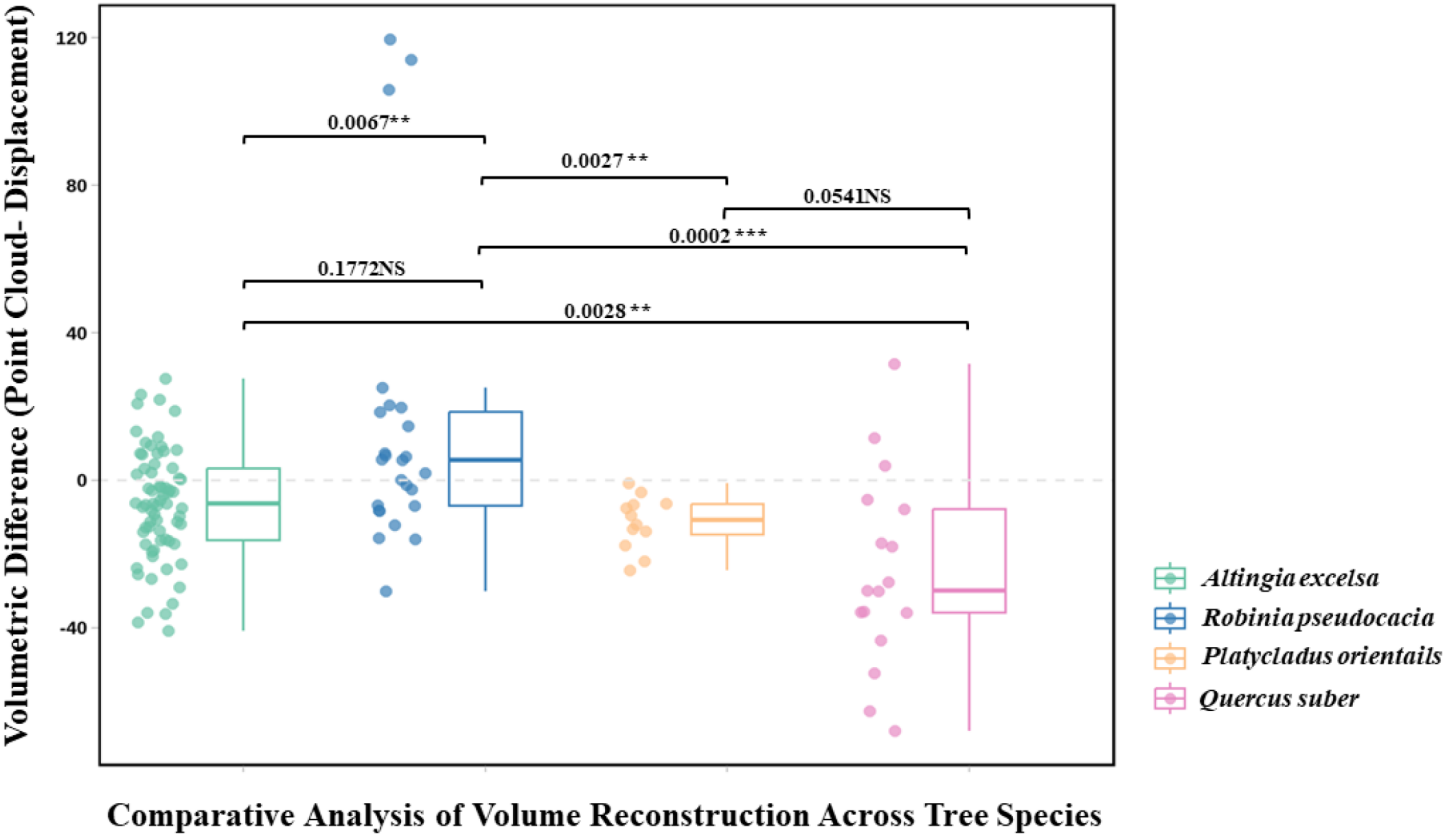
Boxplots showing disc volume estimation errors across different tree species, along with the results of Mann–Whitney U tests to assess statistical differences between groups.

Based on the non-parametric Kruskal-Wallis test (**Figure 8**), this study evaluated the significance of measurement differences among the four tree species, revealing significant differences in measurements between species (H = 21.1606, p = 0.0001). This finding suggests that the influence of species differences on measurement methods should be considered in practical applications. The non-parametric test, which does not require assumptions of normality or homogeneity of variances, was suitable for this study given the significant variance heterogeneity among groups. Pairwise comparisons using the Mann-Whitney U test further clarified specific differences between species. **Figure 8** presents a comparative analysis of Trunk disk sample deviations between point cloud-based reconstruction and water displacement measurements across four representative tree species(*Altingia excelsa, Robinia pseudoacacia, Platycladus orientalis, and Quercus suber*), clearly highlighting species-specific patterns. Data analysis indicated significant statistical differences in trunk volume estimation errors among species (p < 0.01). *Platycladus orientalis* exhibited the highest estimation accuracy, with a narrow boxplot concentrated near zero, showing only a slight and consistent underestimation trend. *Altingia excelsa* followed, with a relatively concentrated error distribution and a median close to zero, indicating overall accurate trunk volume estimates with a slight underestimation bias. *Robinia pseudoacacia* displayed a more dispersed error distribution, with a median significantly above zero, reflecting a systematic overestimation trend. *Quercus suber* showed the lowest trunk volume estimation accuracy, with its boxplot distinctly skewed toward negative values, demonstrating a significant and consistent underestimation pattern. Pairwise Mann-Whitney U comparisons revealed more detailed difference patterns, indicating that *Robinia pseudoacacia* exhibited significant differences from all other species.

## 4 Discussion

This study demonstrates that terrestrial laser scanning (TLS) exhibits strong overall performance in tree trunk volume estimation, fully meeting the expectations for accurate trunk volume estimation. Our regression analysis reveals a slight systematic bias in TLS-based disc trunk estimates compared to water displacement measurements: slight overestimation for small-trunk volume samples and slight underestimation for large-trunk volume samples. To address the issue of missing bottom surface data in disc sample point clouds, the bottom surface filling algorithm developed in this study, based on the point cloud projection principle, can be employed to ensure the model completeness.

Through the Kruskal-Wallis test, the study identified significant differences in measurement accuracy among tree species (H = 21.1606, p = 0.0001). Pairwise comparisons using the Mann-Whitney U test further clarified specific difference patterns among species, with *Robinia pseudoacacia* showing significant differences from all other species. To explore the reason for this, systematic analysis of the disc samples were carried out, and revealed that tree species characteristics influence the accuracy of TLS trunk volume estimation primarily through several factors, with bark characteristics being the most dominant.

### 4.1 Impact of bark characteristics

In the disc samples, the bark occupies the peripheral edge region, and differences in growth environment, genetic characteristics, and adaptive strategies among tree species result in distinct bark structures and surface properties (Pasztory et al., 2016). These characteristics directly affect the quality of laser reflection signals and the accuracy of point cloud data. Consequently, variations in bark properties significantly influence the precise capture of disc sample edge contours, thereby substantially impacting trunk volume estimation results. As the outermost layer directly interacting with the near-infrared laser, the bark surface affects the quality of laser reflection signals, making it the primary factor influencing the accuracy of laser scanning trunk volume estimation.

Among all studied tree species, *Quercus suber* exhibited the largest trunk volume estimation error (mean relative error of 12.98%), which is closely associated with its unique bark characteristics. *Quercus suber* has thick, rough bark with a pronounced blocky exfoliation pattern, leading to complex scattering of incident laser beams during reflection (Pereira, 2011; Reed and Simon, 1979). When near-infrared laser beams strike the rough bark surface, the light scatters in multiple directions rather than undergoing specular reflection, resulting in diffuse reflection that reduces the intensity and accuracy of the returned signal (Malhi et al., 2018). Deep grooves and cracks on the bark surface further cause multiple reflections and shadowing effects, leading to “voids” or uneven density regions in the point cloud data. These factors collectively contribute to a significant underestimation trend in the trunk volume estimation of *Quercus suber* disc samples (mean bias of -24.83) and greater variability (standard deviation of bias of 25.32).

The bark characteristics of *Robinia pseudoacacia* are intermediate between smooth and rough, exhibiting moderately deep longitudinal cracks but less pronounced blocky exfoliation compared to *Quercus suber* (Keresztesi, 1988). The trunk volume estimation accuracy for *Robinia pseudoacacia* samples (mean relative error of 9.58%) was superior to that of *Quercus suber* but inferior to *Platycladus orientalis*. In contrast, *Altingia excelsa* and *Platycladus orientalis* have relatively smooth bark with more uniform surface structures, providing more stable laser reflection properties (Ickert-Bond and Wen, 2006; Li et al., 2016). Although *Altingia excelsa* exhibits shallow longitudinal wrinkles, its high surface flatness results in a trunk volume estimation accuracy (mean relative error of 9.65%) comparable to that of *Robinia pseudoacacia*. Notably, *Platycladus orientalis* has the smoothest and most finely textured bark, with a regular pattern that minimizes laser signal scattering and interference, leading to significantly higher trunk volume estimation accuracy than other species (mean relative error of 7.66%). Therefore, in practical applications, scanning parameters and data processing strategies should be adjusted according to the bark characteristics of different tree species. For species with rough bark, additional scan positions should be added to improve the accuracy and reliability of trunk volume estimation.

### 4.2 Effects of wood structural characteristics

The cross-sectional characteristics of disc samples further amplify the impact of internal wood structure on laser scanning accuracy. For larger-diameter discs, the cut surface contributes more significantly to trunk volume calculations. Consequently, differences in wood structure characteristics primarily affect the uniformity and reflectivity of the disc’s cut surface, directly influencing the accurate capture of the cross-sectional contour and significantly impacting trunk volume estimation results.

*Robinia pseudoacacia* and *Quercus suber*, as ring-porous species, exhibit markedly different laser reflection characteristics compared to diffuse-porous species in disc samples. The characteristic annual ring structure of ring-porous wood, with large earlywood vessels and dense latewood vessels, creates a pronounced density gradient and uneven surface (Schoch et al., 2004). This structure results in highly variable reflection signals during laser scanning, increasing the noise level in point cloud data (Jian et al., 2011). As a typical ring-porous species, *Robinia pseudoacacia* shows the greatest variability in estimation results, with a tendency for overestimation. This suggests that micro-depressions formed by large vessels increase the surface area, leading to an overestimation of actual trunk volume.

In contrast, diffuse-porous species such as *Altingia excelsa* have relatively uniform vessel distribution and stable wood structure, resulting in higher cut surface flatness and more consistent laser reflection signals (Schoch et al., 2004). Coniferous species like P*latycladus orientalis*, lacking true vessel structures, possess more homogeneous wood tissue and smoother cut surfaces, yielding the highest-quality laser scanning data and the best trunk volume estimation accuracy. These findings align with Pretzsch et al. (2017) (Pretzsch et al., 2017), who reported the influence of wood structure on remote sensing data quality.

### 4.3 Limitations and outlooks

Given the limitations of this study, several shortcomings remain: First, the study is based on disc samples, yet the complex and diverse morphology of trees requires further validation before applications can be extended from discs to whole trees. Although this study focused on disc-level volume estimation, its direct applicability to full-stem or whole-tree volume estimation remains limited. Future research is encouraged to extend the approach by reconstructing full trunk volumes for at least a subset of sample trees using established TLS modeling frameworks such as TreeQSM, AdQSM, RCT, or SimpleForest. Such efforts would allow cross-validation between disc-based and whole-tree estimates, enhancing the method’s robustness and expanding its utility for forest inventory and biomass assessments. Moreover, although this study developed a customized 3D reconstruction pipeline based on surface filling and Poisson reconstruction, it lacks benchmarking against widely used volume estimation methods such as QSM, convex hull, and voxel-based approaches. Including both quantitative and qualitative comparisons in future work will help readers better evaluate the relative performance of the proposed method.

Second, the sample types in this study are limited, failing to encompass a broader spectrum of tree species. In particular, the number of samples was uneven among species, with 10 *Altingia excelsa trees* collected from the Puwen area, but only 3 *Robinia pseudoacacia*, 1 *Platycladus orientalis*, and 3 *Quercus suber trees* from the Xiaolangdi area, which may influence the statistical robustness of species-specific comparisons.

Future research should focus on advancing in the following directions: exploring the integration of artificial intelligence techniques with multi-source data fusion to further mitigate occlusion and noise interference in complex forest environments, thereby validating and enhancing estimation accuracy and efficiency at the individual tree and stand scales; and investigating non-destructive methods for estimating tree density. Accurate estimation of aboveground forest biomass based on three-dimensional trunk volume and density is a critical direction for advancing precision forestry and ecological monitoring. By integrating non-destructive technologies such as terrestrial laser scanning, hyperspectral remote sensing, and multi-frequency microwave instruments, species-specific density prediction models can be developed. Combining these with high-precision three-dimensional point cloud trunk volume estimation enables efficient biomass measurement at individual tree and stand scales, reducing ecological disturbance while improving estimation accuracy.

## 5 Conclusions

This study validated the feasibility of using TLS data for accurate and non-destructive estimation of tree disc volume. To address the issue of missing bottom surface data in TLS scans, a bottom surface completion algorithm based on the point cloud projection principle was proposed. Despite a slight but consistent underestimation bias, the TLS-derived trunk volume estimates showed a strong linear correlation with the true trunk volumes measured by the water displacement method (R² = 0.940, rRMSE = 14.92%). At the species level, bark and wood characteristics were found to significantly influence the accuracy of TLS-based trunk volume estimation. *Platycladus orientalis*, with its smooth bark and homogeneous wood structure, provided ideal conditions for laser scanning and achieved the highest estimation accuracy (rRMSE = 4.37%). *Altingia excelsa* showed moderate accuracy (rRMSE = 10.23%) with a slight and stable underestimation trend. *Robinia pseudoacacia* exhibited greater variability and a tendency toward overestimation, which may be attributed to its relatively rough bark and ring-porous wood structure. *Quercus suber* had the lowest estimation accuracy, with a pronounced underestimation trend in larger-trunk volume samples, likely due to its thick, rugged bark and high wood density.In addition, the study recommends optimizing scanning parameters and data processing strategies based on species-specific traits. For example, increasing scan angles for species with rough bark and applying curvature-adaptive surface fitting methods can further enhance measurement accuracy. Overall, TLS technology provides a robust and practical solution for high-precision trunk volume estimation across multiple tree species, offering promising applications in forest inventory and precision forestry.

## Data Availability

The original contributions presented in the study are included in the article/supplementary material. Further inquiries can be directed to the corresponding authors.
